# The Potential of Transforming Growth Factor-beta Inhibitor and Vascular Endothelial Growth Factor Inhibitor as Therapeutic Agents for Uterine Leiomyoma

**DOI:** 10.7150/ijms.75203

**Published:** 2022-10-03

**Authors:** Jung Yoon Park, Boah Chae, Mee-Ran Kim

**Affiliations:** Department of Obstetrics and Gynecology, Seoul St. Mary's Hospital, College of Medicine, The Catholic University of Korea.

**Keywords:** leiomyoma, axitinib, infertility, ulipristal acetate, mifepristone

## Abstract

**Background:** Uterine leiomyoma is the most common benign tumor in women of reproductive age, and it can cause infertility. The growth of uterine leiomyoma is mediated by various steroids and growth factors. The purpose of this study was to evaluate the expression of various growth factors in uterine leiomyoma. Additionally, comparing the effects of existing medication and specific growth factor inhibitors on leiomyoma and the normal myometrium, we aimed to see the potential of transforming growth factor-beta (TGF-β) inhibitors and vascular endothelial growth factor (VEGF) inhibitors as therapeutic drugs for uterine leiomyoma.

**Methods:** This *in vitro* study included uterine leiomyoma samples from 12 patients who underwent hysterectomy by laparoscopy or laparotomy at Seoul St. Mary's Hospital between May 2016 and March 2018. Normal myometrium and uterine leiomyoma tissue were obtained from each patient and the expression of growth factors was compared using immunohistochemical staining. After the primary culture of normal myometrial and leiomyoma cells, cell viability was evaluated following treatment with 100 nM ulipristal acetate (UPA) and mifepristone for 48 h. Western blot analysis was performed to determine the protein expression of each growth factor. Cell viability was determined following treatment with a 10-µM TGF-β inhibitor (LY364947) and a 5-µM VEGF inhibitor (axitinib) for 24 h in cultured normal myometrium and leiomyoma cells.

**Results:** Immunohistochemical staining revealed the significantly higher intensity of TGF-β and VEGF in the leiomyoma tissue than in the normal myometrium (P < 0.05). Mifepristone treatment decreased VEGF expression by 62% in the leiomyoma cells (P < 0.05). According to the cell counting kit-8 (CCK-8) assay, cell viability was decreased after UPA, mifepristone, TGF-β1 inhibitor, and VEGF inhibitor treatments in the normal myometrium and leiomyoma tissue. The effects of the TGF-β1 inhibitor significantly differed between normal myometrium and leiomyoma tissue, with a greater decrease in cell survival in the leiomyoma tissue (P < 0.05). Post-hoc analysis showed that the TGF-β1 and VEGF inhibitors had a greater inhibitory effect on leiomyoma tissue compared with that of UPA.

**Conclusion:** TGF-β and VEGF inhibitors significantly decreased the viability of uterine leiomyoma cells, showing stronger effects than the conventional drug, UPA. TGF-β1 inhibitors affect both leiomyoma tissue and the normal uterus; thus, targeted local treatment rather than systemic treatment should be considered.

## Introduction

Uterine leiomyomas are the most common benign tumors in women of childbearing age, causing excessive menstruation and pressure symptoms 1. This condition can cause infertility, miscarriage, and complications during pregnancy 2, 3. The prevalence of leiomyomas in infertile women is 5%-10% 4, 5. A meta-analysis published in 2009 based on data from 18 studies showed that regardless of localization, the presence of leiomyoma results in a significant decrease in fertility rates and an increase in miscarriage rates 6. Uterine leiomyomas and infertility are closely related to age 7; the prevalence of leiomyoma increases with age, which is important because the age at which women bear children has gradually increased 7. Despite the importance of fertility and the quality of life of women, treatments for leiomyoma are limited and have not been widely examined. In addition to surgical treatment, only a few medical treatments are available 1. Such medications exert therapeutic effects by regulating ovarian steroid signaling but show limitations, such as long-term side effects, resulting in hypo-estrogenemia 8. Thus, new targeted therapies are needed. Recently, studies on the early disease stages suggested that leiomyoma development is a multi-step process involving various cytokines and various growth factors, although the molecular mechanisms are not completely understood. The process is considered to involve insulin-like growth factor-1 (IGF-1), platelet-derived growth factor, vascular endothelial growth factor (VEGF), epidermal growth factor (EGF), and basic fibroblast growth factor 9, 10. The purpose of this study was to compare the expression of the representative growth factors in patients with uterine leiomyoma and to compare the effect of the currently used medications with growth factor inhibitors on myoma tissue and the normal myometrium.

## Materials and methods

### Subjects and tissue collection

Twelve uterine leiomyoma tissues were obtained from women who underwent hysterectomy by laparoscopy or laparotomy at Seoul St. Mary's Hospital from May 2016 to March 2018. The patients were aged between 38 and 52 years, with a mean age of 44.5 years. All patients had a menstrual cycle with symptomatic leiomyoma, such as abnormal uterine bleeding, dysmenorrhea, and pressure symptoms. Uterine leiomyoma is diagnosed preoperatively by ultrasonography and magnetic resonance imaging and histologically confirmed after surgery. Eight patients had multiple leiomyomas and four had single leiomyoma. For all patients, normal uterine myometrium and leiomyoma tissues were collected from the uterus parts removed after hysterectomy. Two of the 12 tissue samples obtained from the patients were discarded because they did not reach sufficient saturation. Informed consent was obtained from each patient before surgery for the use of uterine tissues. All tissues were collected in accordance with the guidelines of the Declaration of Helsinki, and approval for the use of uterine leiomyoma was granted by the Institutional Review Board of Seoul St. Mary's Hospital (No. KC 12TNSI0822).

### Cell culture

Uterine leiomyoma tissues were dissected from the uterus and washed with PBS. The tissues were minced into small pieces and digested in 30 mg/mL collagenase IV and 4 mg DNase I at 37 °C for 3 h. The leiomyoma cells were collected by centrifugation at 1,000*g* for 3 min and washed three times with phosphate buffered saline (PBS). Isolated leiomyoma cells were cultured in 100-mm^2^ culture dishes at 37 °C and 5% CO_2_ in a humidified atmosphere in phenol red-free Dulbecco's modified Eagle's medium/F12 (Gibco Life Technologies, Grand Island, NY, USA) supplemented with 10% fetal bovine serum and 100 U/mL penicillin (Gibco Life Technologies, Grand Island, NY, USA). The morphological characteristics were observed daily under an inverted microscope with the medium changed every 2 days. When the cells reached saturation, they were isolated using trypsin-EDTA (Gibco Life Technologies, Grand Island, NY, USA).

### Drug treatment

Ulipristal acetate (UPA) (100 nM; HRA Pharma, Paris, France), mifepristone (RU486; Sigma-Aldrich, St. Louis, MO, USA), LY364947 (inhibitor of TGF-β; Cat. #2718, Tocris Co., Ellisville, MO, USA), and axitinib (inhibitor of VEGF; Cat. #4350, Tocris Co.) were used to treat the cells for 48 h.

### Immunohistochemistry staining

For pathological examination of resected specimens, the tissues were fixed with 10% formalin for 24 h at 25 °C and embedded in paraffin. Formalin-fixed and paraffin-embedded specimens were cut into 5-µm-thick sections. The tissues were rehydrated using xylene three times for 10 min and a series of graded ethanol (absolute ethanol twice, 90% ethanol, 80% ethanol, and 70% ethanol each for 5 min) at 25 °C. After washing with water for 5 min, the sections were permeabilized using 3% H_2_O_2_ in methanol for 10 min at 25 °C and washed again with water for 5 min. For antigen retrieval, the sections were immersed in citrate buffer (pH 9.0; DAKO S2367, Glostrup, Denmark), boiled for 10 min, cooled to 25 °C for 20 min on ice, washed with water, and blocked with protein block serum-free (DAKO X0909) for 30 min at 25 °C. The tissues were incubated with anti-EGF (ab9695, Abcam), TGF-β (ab92486, Abcam), VEGF-A (ab1316, Abcam), and IGF-1 (ab9572, Abcam, Cambridge, UK) antibodies (1:500) overnight at 4 °C. The sections were washed with wash buffer (DAKO S3006) prior to incubation with DAKO REAL^TM^ ENVISION/HRP, rabbit/mouse (DAKO K5007) for 30 min at 25 °C.

### Western blot immunoassay

Proteins were extracted from the cultured uterine leiomyoma cells. After drug treatment, the cells were lysed at 4 °C for 30 min in lysis buffer. The lysates were centrifuged at 1,000*g* for 30 min at 4 °C, and the supernatants were collected. The protein content in the supernatants was determined using the BCA assay (cat 23227). Each 50-µg aliquot of protein extracted from cultured cells was separated using a NuPAGE Novex 4%-12% Bis-Tris Gel (Invitrogen Life Technologies, Carlsbad, CA, USA) under reducing conditions at 200 V for 50 min. The proteins were electrophoretically transferred from the gels onto polyvinylidene fluoride transfer membranes (Amersham, Piscataway, NJ, USA). The blots were exposed overnight at 4 °C to primary antibodies: a mouse monoclonal antibody to VEGF (ab1316, Abcam) and a rabbit polyclonal antibody to EGF (ab9695, Abcam), IGF-1 (ab9572, Abcam), or TGF-β (ab92486, Abcam) at a dilution of 1:200. The blots were washed three times with Tris-buffered saline containing 1X Tween 20. The membranes were incubated for 1 h with anti-mouse or anti-rabbit secondary antibodies (Santa Cruz Biotechnology, Dallas, TX, USA) diluted to 1:1000 with blocking buffer. Characteristic protein bands were detected using the ECL western blotting system (Amersham). Protein levels on the blots were standardized to the levels of β-actin 1:1000 (sc47778, Santa Cruz Biotechnology). The membranes were visualized by exposure to an X-OMAT film (Eastman Kodak Co., Rochester, NY, USA).

### Cell viability assay

The leiomyoma and normal myometrial cells were seeded into a 96-well plate, incubated for 24 h, and then treated with 100 nM UPA and mifepristone for 48 h at 37 °C and 5% CO_2_. The cells were treated with 10 µM LY364947 (inhibitor of TGF-β; Cat. #2718, Tocris Co., Ellisville, MO, USA) and 5 µM axitinib (inhibitor of VEGF; Cat. #4350, Tocris Co.) for 24 h. Cell counting kit-8 (CCK-8, Dojindo Molecular Technologies, Inc., Kumamoto, Japan) assay was used to determine cell viability according to the manufacturer's instructions.

### Statistical analysis

Statistical software (SPSS 23.0, SPSS, Inc., Chicago, IL, USA) was used for data analysis. For statistical analysis, the chi-square test and one-way analysis of variance were performed. Measured data were expressed as the mean ± standard deviation, and *t*-tests were used to analyze data between the two groups. One-way analysis of variance and Scheffe's post-hoc test were used to compare multiple treatment options. Statistical significance was set at P < 0.05.

## Results

### Histological expression of growth factor and quantification

**Figure 1:**
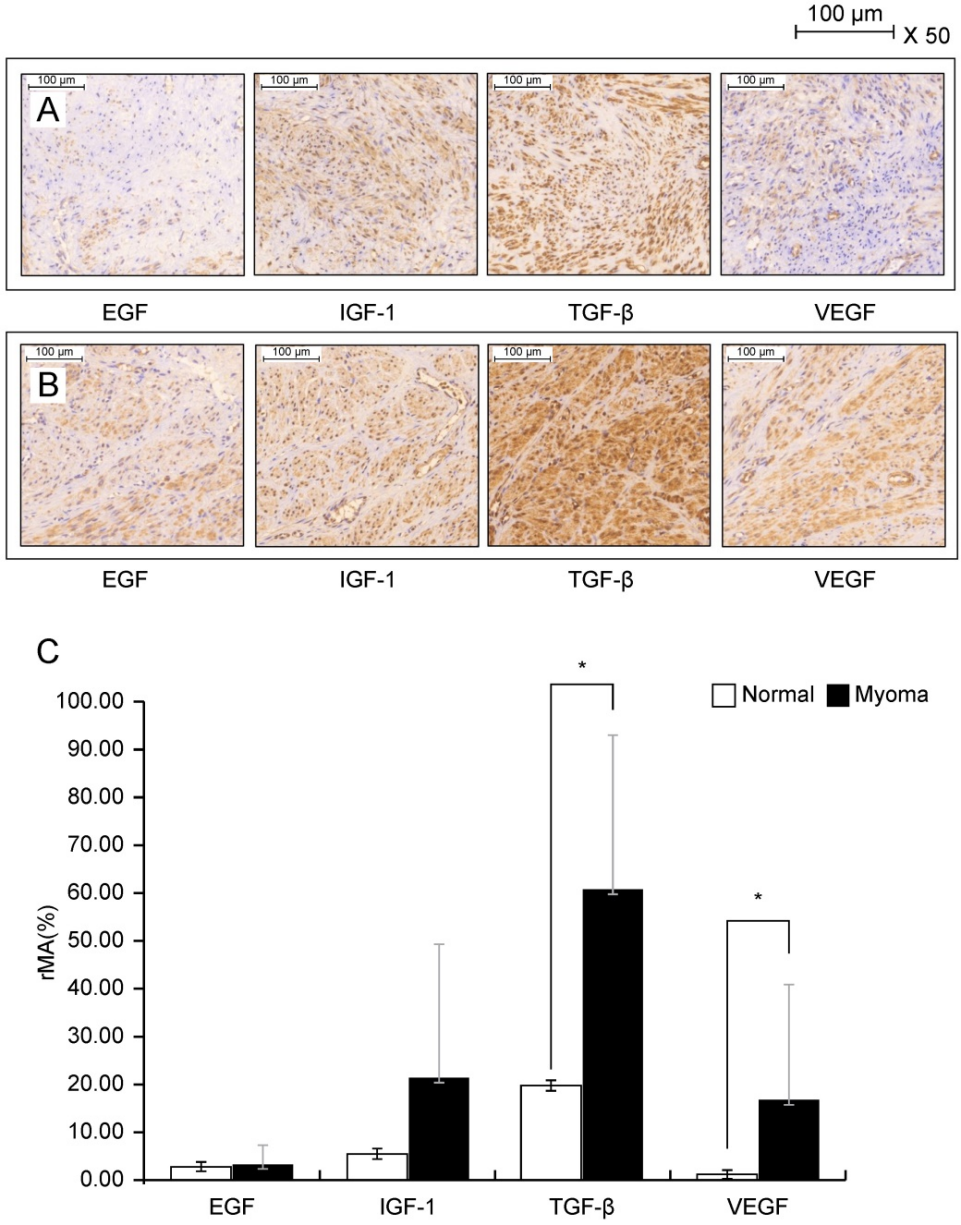
Immunocytochemical staining of IGF-1, 217EGF, TGF-β, and VEGF in the normal myometrium (**A**) and leiomyoma tissue (**B**). Leiomyoma cells are cultured for 24 h at 25 °C and the tissues are incubated overnight with antibodies diluted by 1:500. Bar = 500 µm. The expression (**C**) of each growth factor is calculated using the relative mask area [rMA  =  (MA/FA) × 100]. FA, overall field area (mm^2^); MA, overall mask area (mm^2^), which is the summed area for each detected object in each layer. *P < 0.05 is considered as significant. Abbreviations: EGF, epidermal growth factor; IGF-1, insulin-like growth factor-1; TGF-β, transforming growth factor-beta; VEGF, vascular endothelial growth factor.

Immunohistochemical staining was performed using leiomyoma cells from 12 patients. Figure 1 shows the immunohistochemical staining results of EGF, IGF-1, TGF-β, and VEGF in normal myometrial cells (Fig. 1A, upper panel) and leiomyoma cells (Fig. 1B, middle panel). The expression of growth factors was investigated by calculating the relative mask area (%) in the normal myometrium layer and leiomyoma tissue (Fig. 1C, lower panel). Among the various growth factors, TGF-β and VEGF showed significantly higher staining intensity in leiomyoma tissue than in normal myometrium (P < 0.05). The intensity of TGF-β was 3.1-fold higher in leiomyoma cells than in normal myometrium cells; VEGF showed 14.4-fold higher intensity, whereas EGF and IGF-1 showed no significant difference in normal myometrium and leiomyoma cells.

### Evaluation of growth factor expression on leiomyoma tissue after conventional drug treatment with ulipristal acetate and mifepristone

**Figure 2:**
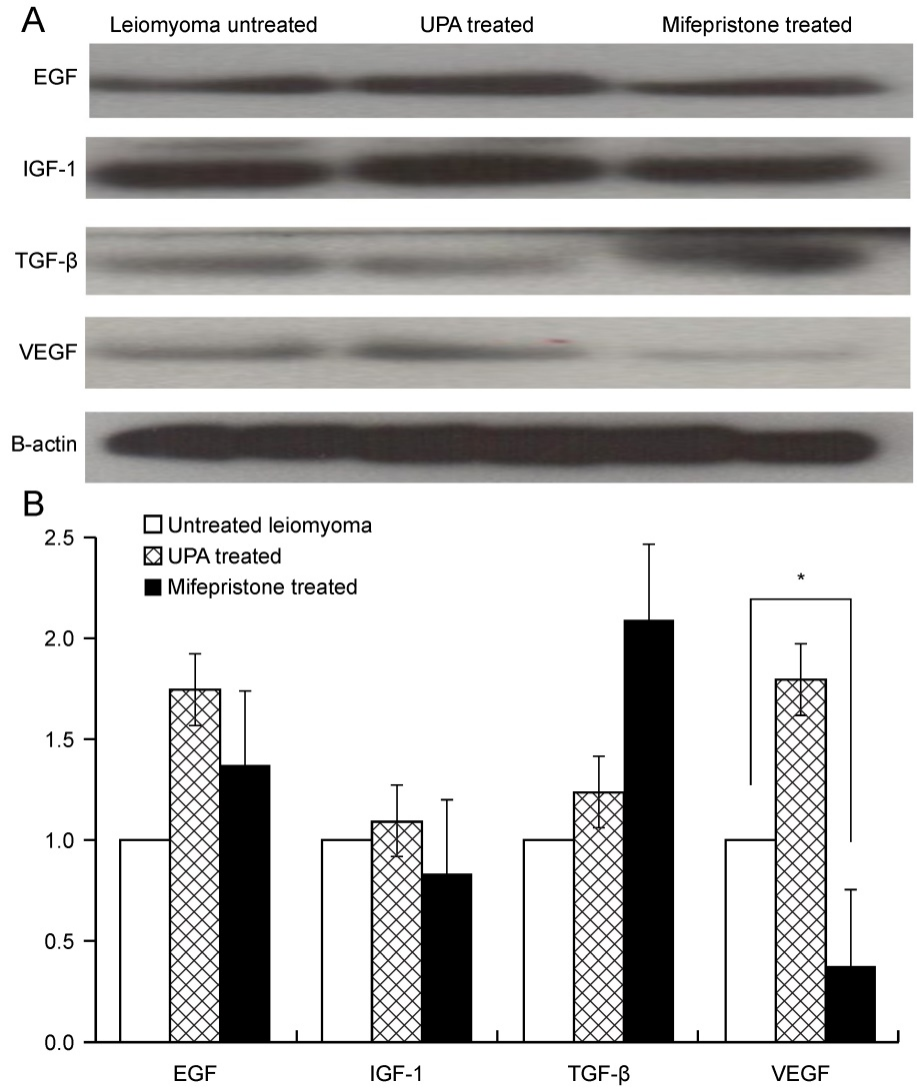
Expression of growth factors EGF, IGF-1, TGF-β, and VEGF in myoma tissue after treatment with UPA and mifepristone. (**A**) Western blotting of growth factors after UPA and 100 nM mifepristone (10-7 M) treatment for 48 h in myoma tissue. (**B**) Densitometric analysis of each growth factor expression. All data are presented as the mean ± standard deviation and were analyzed by using a one-way analysis of variance. *P < 0.05 was considered as significant. Abbreviations: EGF: epidermal growth factor; IGF-1: insulin-like growth factor-1; TGF-β: transforming growth factor-beta; VEGF: vascular endothelial growth factor; UPA: ulipristal acetate.

Figure 2 shows the results of western blotting, which was performed to evaluate the protein expression of growth factors in uterine leiomyoma tissue in the presence or absence of drug treatment for 48 h. The most commonly used medications for leiomyoma treatment, namely UPA and mifepristone, were used to treat uterine leiomyoma tissues. The protein expression of growth factors such as EGF, IGF-1, TGF-β, and VEGF was quantified (Fig. 2A). After mifepristone treatment, VEGF expression was significantly decreased by 62% in uterine leiomyoma cells (P < 0.05). The expression levels of EGF, IGF, TGF-β, and VEGF increased when UPA was used for leiomyoma tissue, but this increase was not statistically significant. In the case of mifepristone, the expression levels of EGF and TGF-β increased and those of IGF and VEGF decreased; however, only the 62% decrease in the expression level of VEGF was statistically significant (P < 0.05) (Fig. 2B).

### Effect on cell viability after TGF-β1 inhibitor, VEGF inhibitor, UPA, and mifepristone on leiomyoma and normal myometrium

To examine the survival of cells in the leiomyoma tissue and normal myometrium after each drug treatment, a CCK-8 assay was performed. Because TGF-β and VEGF were significantly increased in the leiomyoma tissue, we used inhibitors of VEGF and TGF-β1. The cells were also treated with UPA and mifepristone for comparison. Drugs were administered to both the normal myometrium and leiomyoma tissues.

**Figure 3:**
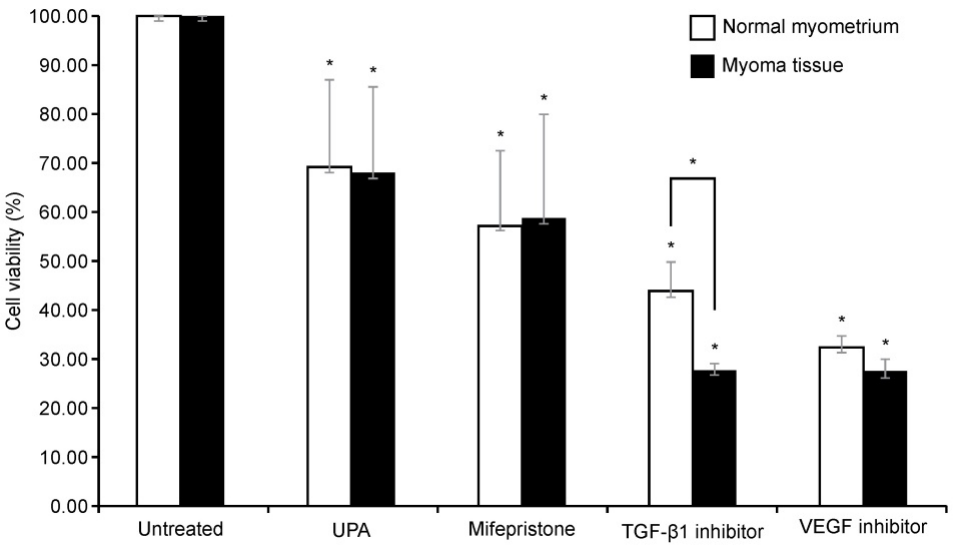
Effect of UPA, mifepristone, TGF-β1 inhibitor, and VEGF inhibitor treatment in normal uterine myometrium and leiomyoma tissue. The normal myometrial tissue and leiomyoma tissue are treated with 100 nM of UPA and mifepristone for 48 h and 10 µM TGF-β inhibitor and 5 µM VEGF inhibitor for 24 h. CCK-8 assays are performed to evaluate cell survival. Cell viability after UPA, mifepristone, TGF-β1 inhibitor, and VEGF inhibitor treatment are analyzed as a percentage compared to non-drug tissue. All data are presented as the mean ± standard deviation and are analyzed using a one-way analysis of variance and Scheffe's post-hoc test. * P < 0.05 was considered as significant. Abbreviations: EGF: epidermal growth factor; TGF-β: transforming growth factor-beta; VEGF: vascular endothelial growth factor; UPA: ulipristal acetate.

As illustrated in Fig. 3, compared with the untreated group, each drug significantly decreased cell viability in both the normal myometrium tissue and leiomyoma tissue. The viability of uterine leiomyoma cells decreased to 67%, 59%, 27%, and 29% after treatment with UPA, mifepristone, TGF-β1 inhibitor, and VEGF inhibitor, respectively, compared to the untreated control. In the normal myometrium, cell viability decreased to 69%, 57%, 49%, and 35% compared to the control group. Only the TGF-β1 inhibitor resulted in significantly different effects on cell viability in the normal myometrium and leiomyoma tissue, causing a greater decrease in the survival rate of leiomyoma cells. There was no significant difference between the two groups after treatment with the other drugs. Post-hoc analysis revealed that the change in cell viability in leiomyoma was greater following treatment with the VEGF and TGF-β1 inhibitors than following treatment with UPA. In the normal myometrium, cell viability was decreased more by the VEGF inhibitor than by UPA (Table 1).

## Discussion

Uterine leiomyoma is the most common benign tumor in women of childbearing age and causes symptoms such as bleeding or pelvic pain infertility 1. A total survey using data from cohorts of one million patients collected from Korean health insurance data from 2002 to 2013 showed that the prevalence of leiomyoma is increasing in women of all childbearing ages, quadrupling to 2.48% from 2002 to 2013 11. In 2018, the average age of women who gave birth in Korea was 32 years, and the proportion of elderly mothers had increased to 31.8% 12. The prevalence of uterine leiomyoma is expected to gradually increase with the development of imaging equipment for diagnosis, leading to socioeconomic loss as the marriage and childbirth age increases. Hysterectomy is considered the absolute treatment for uterine leiomyoma, but alternative treatments are widely used to preserve fertility and avoid invasive surgery 13.

Gonadotropin-releasing hormone agonists, which are currently widely used for treatment, can reduce the size by 40% and improve symptoms after 3 months of treatment, but the effect is temporary (3-6 months) and used only in limited cases because of their various side effects 14, 15. Treatment with gonadotropin-releasing hormone analogs leads to a rebound in fibroid growth and the loss of bone mineral density when the administration is stopped 16. As another option, a selective progesterone receptor modulator with a tissue-specific effect on the progesterone receptor can be used 17. Mifepristone is the first progesterone receptor antagonist and has been used clinically for more than 25 years 18, 19. Cochrane's review of randomized controlled studies showed a decrease in bleeding and increased quality of life in patients administered mifepristone; however, there was no significant decrease in the leiomyoma volume 20. UPA is widely used as a selective progesterone receptor modulator and was approved by the US FDA in 2010 for treating symptomatic leiomyoma in females of childbearing age. However, side effects such as nausea, vomiting, breast tenderness, headache, and malaise can occur; therefore, this drug can only be used before surgery or for a limited time 21-23. Liver injury necessitating liver transplantation has recently been reported in women treated with UPA 22,23.This has led to the suspension of UPA as a medical therapy for treating uterine fibroids while the European Medicines Agency conducts a review of this liver injury risk 16. The European Medicine Agency safety committee advised that women should stop taking 5 mg UPA and that no new patients should commence treatment until the ongoing review is completed.

The effect of long-term use of current medications is currently being evaluated for PERL 4 and VENUS 2, which are not available to women who are planning to become pregnant because of ovulation inhibition 23, 24. The fertility outcome has not been fully discussed. Growth factor control may prevent the effects of steroid changes to effectively treat long-lasting leiomyomas. Unlike previous studies of the effects of individual growth factors, we compared several growth factors simultaneously. We also compared the effects on cell viability in both leiomyoma tissue and normal myometrium. Our results show that currently used medications decrease cell viability not only in leiomyoma tissue, but also in normal tissues. However, among the growth factor inhibitors used in this study, the TGF-β1 inhibitor showed more specific action towards uterine leiomyoma, demonstrating its potential as a therapeutic agent. Several studies showed that TGF-β not only stimulates smooth muscle cells, but also contributes to the growth of uterine leiomyoma 9, 25, and studies of selected cell lines revealed that this growth factor significantly affects the accumulation of extracellular matrix in uterine leiomyoma 26, 27. Cellular responses to TGF-β are diverse, and several studies have demonstrated its pre-tumorigenic role in various stages of cancer; accordingly, many strategies to suppress TGF-β are being evaluated 28. However, systemic therapy, such as targeting TGF-β signaling, shows some limitations such as cardiovascular toxic side effects and benign tumor formation (Colak and Ten Dijke, 2017). We found that the TGF-β inhibitor affected not only myoma tissue, but also the normal myometrium; therefore, targeted local treatment rather than systemic treatment should be considered. As we only evaluated one concentration of each drug, differences in culture conditions and concentrations should be examined to determine the safety margin for the normal myometrium while treating the leiomyoma tissue.

VEGF is an important growth factor that stimulates the proliferation of vascular endothelial cells. VEGF promotes cell migration and proliferation by binding to associated receptors, thereby increasing angiogenesis. Although uterine fibroids are benign tumors, angiogenesis is critical for the occurrence and development of these fibroids 29. The effect of VEGF on the prognosis of leiomyoma gained attention following a study demonstrating a difference in the expression of VEGF and IGF-1 after uterine artery embolization treatment.

Inhibitors of the growth factors TGF-β1 and VEGF showed greater inhibitory effects on leiomyoma cell viability than conventional drugs, indicating their potential as therapeutic agents for uterine leiomyoma. In addition, as previous studies revealed risks associated with the systemic action of growth factor inhibitors, their use as local treatments could be considered.

## Conclusion

The expression of various growth factors (EGF, IGF-1, TGF-β, and VEGF) was evaluated in uterine leiomyoma tissues. The expression levels of TGF-β and VEGF were 3.1- and 14-fold higher in leiomyoma than in the normal myometrium. Among the leiomyoma treatments used in the clinic, UPA did not significantly alter growth factor expression in leiomyoma tissue, whereas mifepristone significantly decreased the expression of VEGF in leiomyoma tissue.

The TGF-β1 inhibitor caused a more significant change in cell viability in leiomyoma tissue than in the normal myometrium. In the post-hoc test, TGF-β1 inhibitor and VEGF inhibitors tended to have greater inhibitory effects than the conventional drug, UPA. Through this study, the potential of growth-inhibitors as a treatment for leiomyoma was examined, and the possibility as a local treatment could also be considered. It is expected that a safer therapeutic concentration can be found through additional experiments with various concentrations in the future.

## Figures and Tables

**Table 1:** Changes in cell viability of myometrium and leiomyoma tissue after UPA, mifepristone, TGF-β1 inhibitor, and VEGF inhibitor treatment and post-hoc analysis of differences between treatments

	Treatment	Mean	SD	P	Post-hoc test
Normal myometrium	UPA (U)	69.10	17.83	0.03	U>A (Scheffe)
	Mifepristone (M)	57.20	15.24		
	TGF-β1 inhibitor (L)	49.09	10.83		
	VEGF inhibitor (A)	34.74	10.14		
Myoma	UPA (U)	67.81	17.53	0.02	U>L,A (Scheffe)
	Mifepristone (M)	58.59	21.31		
	TGF-β1 inhibitor (L)	26.76	1.30		
	VEGF inhibitor (A)	29.24	7.74		

All data are presented as the mean and standard deviation (SD) and were analyzed by using one-way analysis of variance and Scheffe's post hoc test. Abbreviations: EGF: as epidermal growth factor; TGF-β: transforming growth factor-beta; VEGF: vascular endothelial growth factor; UPA: ulipristal acetate.

## Abbreviations

EGF: epidermal growth factor
TGF-β: transforming growth factor-beta
VEGF: vascular endothelial growth factor
UPA: ulipristal acetate
